# Molecular Detection of Mycobacterium tuberculosis from Stools in Young Children by Use of a Novel Centrifugation-Free Processing Method

**DOI:** 10.1128/JCM.00781-18

**Published:** 2018-08-27

**Authors:** Elisabetta Walters, Lesley Scott, Pamela Nabeta, Anne-Marie Demers, Gary Reubenson, Corné Bosch, Anura David, Marieke van der Zalm, Joshua Havumaki, Megan Palmer, Anneke C. Hesseling, Jabulani Ncayiyana, Wendy Stevens, David Alland, Claudia Denkinger, Padmapriya Banada

**Affiliations:** a Desmond Tutu TB Centre, Stellenbosch University, Paediatrics and Child Health, Faculty of Medicine and Health Sciences, Cape Town, South Africa; b Molecular Medicine and Haematology, School of Pathology, Faculty of Health Sciences, University of the Witwatersrand, Johannesburg, South Africa; c Foundation for Innovative New Diagnostics, Geneva, Switzerland; d Rahima Moosa Mother & Child Hospital, University of the Witwatersrand, Faculty of Health Sciences, Paediatrics and Child Health, Johannesburg, South Africa; e Department of Epidemiology, School of Public Health, University of Michigan, Ann Arbor, Michigan, USA; f Division of Epidemiology & Biostatistics, School of Public Health and Family Medicine, Faculty of Health Sciences, University of Cape Town, Cape Town, South Africa; g Division of Epidemiology & Biostatistics, School of Public Health, Faculty of Health Sciences, University of the Witwatersrand, Johannesburg, South Africa; h Rutgers, New Jersey Medical School, Faculty of Medicine, Newark, New Jersey, USA; i National Priority Program of the National Health Laboratory Service, Johannesburg, South Africa; Virginia Commonwealth University Medical Center

**Keywords:** children, diagnosis, stool, tuberculosis

## Abstract

The microbiological diagnosis of tuberculosis (TB) in children is challenging, as it relies on the collection of relatively invasive specimens by trained health care workers, which is not feasible in many settings. Mycobacterium tuberculosis is detectable from the stools of children using molecular methods, but processing stool specimens is resource intensive. We evaluated a novel, simple, centrifugation-free processing method for stool specimens for use on the Xpert MTB/RIF assay (Xpert), using two different stool masses: 0.6 g and a swab sample. Two hundred eighty children (median age, 15.5 months; 35 [12.5%] HIV infected) with suspected intrathoracic TB were enrolled from two sites in South Africa. Compared to a single Xpert test on respiratory specimens, the sensitivity of Xpert on stools using the 0.6-g and swab samples was 44.4% (95% confidence interval [CI], 13.7 to 78.8%) for both methods, with a specificity of >99%. The combined sensitivities of two stool tests versus the first respiratory Xpert were 70.0% (95% CI, 34.8 to 93.3) and 50.0% (95% CI, 18.7 to 81.3) for the 0.6-g and swab sample, respectively. Retesting stool specimens with nondeterminate Xpert results improved nondeterminate rates from 9.3% to 3.9% and from 8.6% to 4.3% for 0.6-g and swab samples, respectively. Overall, stool Xpert detected 14/94 (14.9%) children who initiated antituberculosis treatment, while respiratory specimens detected 23/94 (24.5%). This stool processing method is well suited for settings with low capacity for respiratory specimen collection. However, the overall sensitivity to detect confirmed and clinical TB was lower than that of respiratory specimens. More sensitive rapid molecular assays are needed to improve the utility of stools for the diagnosis of intrathoracic TB in children from resource-limited settings.

## Abstract (precis)

The microbiological diagnosis of tuberculosis (TB) in children is challenging, as it relies on the collection of relatively invasive specimens by trained health care workers, which is not feasible in many settings. Mycobacterium tuberculosis is detectable from the stools of children using molecular methods, but processing stool specimens is resource intensive.

## INTRODUCTION

Tuberculosis (TB) is a leading cause of death in children globally. Data from a recent modeling study calculated that TB ranked as one of the top 5 killers of young children, with an estimated 191,000 annual deaths in children <5 years old (1). The majority (96%) of deaths occurred in children who did not receive treatment (1). The substantial challenges in confirming TB in children contribute to underdetection and lack of access to treatment.

Some of the current barriers to diagnosing pulmonary (intrathoracic) TB (PTB), particularly in young children, include difficulties in obtaining adequate specimens for microbiological confirmation (2) and the low bacterial load in pulmonary secretions. Although culture remains the gold standard for TB diagnosis, it has low sensitivity (30 to 40%) for paucibacillary pediatric TB (3, 4) and a long turnaround time, requires well-equipped laboratory infrastructure, and is prone to contamination. In some resource-constrained settings, these factors limit the feasibility of culture-based diagnosis in children. Molecular tests like the Xpert MTB/RIF assay (Xpert) (Cepheid, Sunnyvale, CA) have become widely used, even in low-resourced settings. Although the sensitivity of Xpert is lower than that of culture in children (estimated 67% for diagnosis of PTB) (5), the assay is fully automated, and the rapidity of results and need for minimal processing before testing, while also informing drug susceptibility status for rifampin, makes Xpert an attractive alternative to culture. With the development of the Xpert ultra, with improved sensitivity compared to that of the Xpert (6), the use of this technology is likely to expand further.

**FIG 1:**
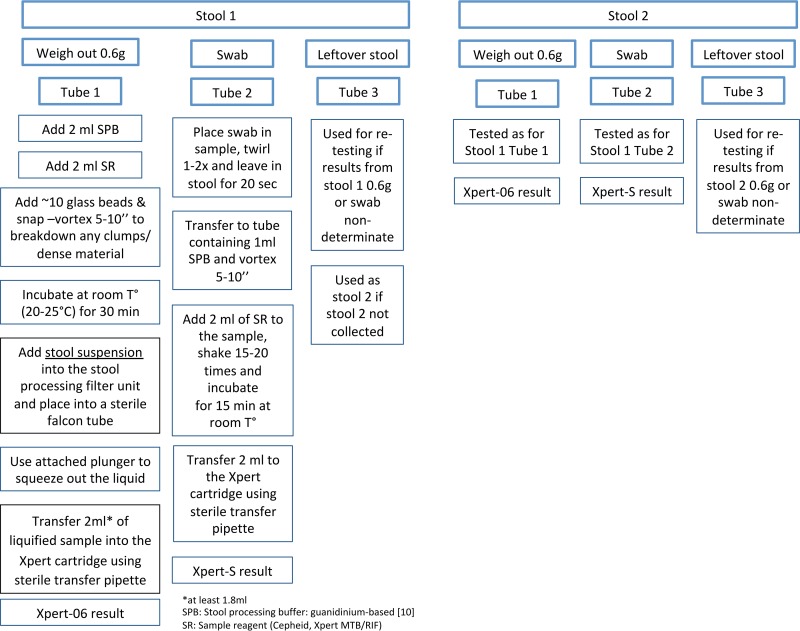
Stool specimen preparation flow diagram and outline of stool processing methods.

The use of alternative, less invasive specimens, including stool samples, which are potentially easier to collect than gastric aspirates (GA), induced sputum (IS), and nasopharyngeal aspirates (NPA), may encourage better attempts at microbiological confirmation of TB, particularly in young children. A number of studies have evaluated the Xpert used on stool for the diagnosis of pediatric PTB, reporting sensitivities of 47 to 75% compared to those of culture and Xpert on respiratory specimens (7–9). However, the procedure for stool processing to date has been labor intensive and time consuming, requiring multiple steps and centrifugation (7–9). A new stool processing (SP) method was developed by Banada et al. at the Alland laboratory (Rutgers Biomedical and Health Sciences, Newark, NJ), for easier, more rapid processing of pediatric stool specimens for Xpert testing (Fig. 1) (10). Initial proof-of-concept work confirmed that Mycobacterium tuberculosis was detectable by Xpert on pediatric stool specimens processed by this method.

In this hospital-based study, we evaluated the SP method for Xpert testing using different initial stool masses, i.e., 0.6-g and swab samples (referred to below as Xpert-06 [Xpert on 0.6 g stool] and Xpert-S [Xpert on stool swab]), respectively, for the diagnosis of PTB in children investigated at two sites in South Africa. We report the diagnostic yield of this method, the incremental value of testing a second stool sample, and the impact of pretest probability and influence of a reference standard when evaluating the diagnostic performance of this new testing method for pediatric PTB.

(This work forms part of the body of work toward a Ph.D. degree for E. Walters.)

## MATERIALS AND METHODS

### Study setting, population, and eligibility.

The study was conducted at two public referral hospitals offering general and specialized pediatric care. At site 1, the Rahima Moosa Mother and Child Hospital in Johannesburg, the study was part of routine clinical care; clinical and microbiological investigations, except for stool testing, followed local practice. All children identified by the attending medical staff as requiring investigation for suspected PTB were eligible for enrollment, including children with chronic or recurrent respiratory symptoms, severe or complicated pneumonia, severe or unexplained malnutrition, and extrapulmonary symptoms compatible with TB in conjunction with abnormal chest radiology. At site 2, Desmond Tutu TB Centre, enrolling at Tygerberg and Karl Bremer Hospitals in Cape Town, an ongoing prospective diagnostic TB study (parent study) supported this work as a substudy. The parent study had well-defined eligibility criteria, a study-specific specimen collection schedule, and protocols for mycobacterial testing and also included an evaluation of Xpert on stools, different from this substudy (7). Children were eligible to be part of both the parent and substudy; therefore, a number of children enrolled in this substudy have been described previously (7). Eligibility was based on any well-defined symptom of PTB (11) or, alternatively, a short history of cough but also other evidence suggestive of TB, including TB exposure in the preceding 12 months, a reactive Mantoux tuberculin skin test (TST), or chest X-ray (CXR) suggestive of TB (using a standard reading form).

Children were excluded if they had received more than one dose of antituberculosis treatment within 60 days prior to enrollment, had extrathoracic TB without concurrent suspected intrathoracic PTB, were clinically unstable, or lived remotely with no access to transport for follow-up visits.

### Study procedures.

Clinical evaluation included information on any previous TB episodes, current/recent (past 12 months) TB exposure, and TST if available. HIV testing followed local guidelines: at site 1, in children <18 months of age, the mother was tested using two rapid HIV tests from different manufacturers. If these were negative, the child was classified as “HIV unexposed” and not tested unless clinically indicated. If the two rapid HIV tests were discordant, an HIV antibody test (enzyme-linked immunosorbent assay [ELISA]) was performed. In children of HIV-infected mothers and in all children >18 months of age, a confirmatory test was completed on the child: HIV DNA PCR if <18 months old or HIV ELISA for children ≥18 months old. At site 2, all children were initially tested by either an HIV DNA PCR or ELISA as described for site 1, unless phlebotomy was insufficient, in which case a rapid HIV test was performed. This was followed by a confirmatory test if positive.

CXRs (frontal and lateral films) were completed and evaluated retrospectively by independent blinded experts, reporting according to a standardized format. Severe TB was defined as any of the following: lymph node disease with airway compression, any cavitation, miliary TB, or expansile pneumonia (12).

### Specimen collection for TB testing. (i) Respiratory specimens.

At site 1, health care workers collected an expectorated sputum (ES) or IS specimen, depending on the child's ability to expectorate spontaneously. In addition, a GA was collected as a second respiratory specimen in a subset of children <5 years of age. Each single specimen was collected in two specimen containers: one specimen was processed onsite using Xpert; the second was sent to the nearby National Health Laboratory Service (NHLS) in Braamfontein, Johannesburg, for decontamination and concentration using *N*-acetyl-l-cysteine (NALC)-NaOH (final NaOH concentration, 1.25%) for TB microscopy and liquid culture (mycobacterial growth indicator tube [MGIT]).

At site 2, study personnel collected a minimum of two respiratory specimens on two separate occasions (4 h apart if on the same day or on two consecutive days). For children unable to expectorate (typically <5 years old), samples included a GA and an IS (with nasopharyngeal suctioning). For children able to expectorate, an early morning ES and an IS (with expectoration) were collected. Samples were processed at NHLS Tygerberg, using NALC-NaOH (final NaOH concentration, 1.25%) (7) and tested using fluorescent smear microscopy, MGIT, and the Xpert test.

At both sites, acid-fast-bacillus (AFB)-positive cultures were identified as M. tuberculosis complex by using the MTBDR*plus* line probe assay (LPA). Second-line phenotypic drug susceptibility testing (DST) was performed at site 1 if resistance to any of the first-line drugs was reported and at site 2 if rifampin resistance was detected on LPA.

### (ii) Stool specimens.

At both sites, a minimum of one and up to two stool specimens were collected from each participant according to study standard operating procedures, no more than 48 h apart and within 7 days of collection of respiratory specimens. Stool was collected from the diaper in young children or from cling wrap fitted over the toilet seat in toilet-trained children. Infants with liquid stools had nylon waterproof material fitted under the diaper for collection of stool. Stool was transferred into a 25-ml fecal cup with the included spoon (PLPS109148; LASEC, Cape Town, South Africa). At site 1, stool specimens were tested immediately onsite in a point-of-care laboratory adjacent to the children's ward; therefore, no storage or transport was required. Specimens at site 2 were transported in a cooler box to the laboratory as soon as possible after collection (same day) and were refrigerated at 2 to 8°C until processing (within 72 h of collection). Caregivers collecting stool at home were instructed to keep the specimens refrigerated until they were collected by the study team.

Collection of a stool mass of at least 2 g (6 scoops using the spoon fitted onto the cap of the fecal cup) was recommended, to allow for the two volumes to be tested (swab and 0.6 g). Each of the two stool specimens collected was tested individually. However, if only one stool specimen was available, the same specimen could be used for the second analysis if the residual volume was sufficient. Stool specimens were tested onsite using the Xpert SP protocol (Fig. 1). The laboratory technician recorded the macroscopic appearance of each stool specimen in a standard form before testing.

### Treatment and follow-up.

The results of the Xpert test (respiratory specimens and stool) and MGIT culture, including DST, were reported to the attending clinicians. Attending clinicians decided on antituberculosis treatment according to clinical guidelines and local standard of care. These treatment decisions were documented by the study team.

A follow-up visit 8 to 10 weeks after enrollment was conducted. A study clinician with access to all laboratory results assessed the response to antituberculosis treatment based on symptoms, signs, weight gain, and CXR. In children not initiated on antituberculosis treatment, symptom resolution was assessed. Children were then classified according to international consensus clinical case definitions (13).

### Statistical analysis.

Descriptive analyses were completed using median values and interquartile ranges for continuous data with nonnormal distribution and proportions for discrete data.

The index test under evaluation, the stool Xpert, was compared to two reference standards: (i) a single Xpert and (ii) a single liquid culture, each on a respiratory specimen. The overall detection by stool Xpert for any method was compared to the clinical case definitions for diagnostic studies (13), where confirmed TB was defined as any child confirmed by Xpert or culture on any respiratory specimen: this analysis follows Standards for Reporting of Diagnostic Accuracy (STARD) guidelines (14), as the clinical case definitions incorporate the gold standard for pulmonary TB by defining “confirmed TB” as TB confirmed by Xpert or culture on any respiratory specimens. In addition, the sensitivity of testing one stool by both 0.6-g and swab sample methods was compared to the sensitivity of a single respiratory culture for the detection of (i) confirmed TB and (ii) clinical TB, defined as the clinician's decision to treat for TB.

Nondeterminate (invalid/error/no result) results for Xpert on respiratory and stool specimens were repeated on the same specimen if sufficient specimen was available. The final result used for the diagnostic analysis was the result of the second test, if done (for nondeterminate), or of the initial test, if the test was not repeated. Stool and respiratory specimens with a final nondeterminate Xpert result after repeat testing, as well as respiratory specimens with contaminated culture results, were excluded from analysis.

The sensitivity, specificity, and positive and negative predictive values were calculated individually for Xpert-06 and Xpert-S. The additional yield of a second stool test (using a second stool specimen if available or by retesting the first stool if the residual volume was sufficient) for each method was determined and was calculated as the percent increase in detection above that of the first stool test. The combined yield of two stool specimens (or two stool tests if one stool was used) was calculated.

Univariate analysis was used to identify factors associated with a positive stool Xpert result. As the outcome was only present in 14 children, the multivariable model only included two variables. For multivariable regression and comparative analyses, odds ratios and 95% confidence intervals (CIs) are reported, along with *P* values; a *P* value of <0.05 was considered statistically significant. Analyses were generated using Stata 14.0 special edition software (Stata Statistical Software, release 14, 2015; StataCorp LP, College Station, TX, USA). STARD guidelines were used for analysis and reporting (14).

### Ethical considerations.

The study was approved by the University of the Witwatersrand Human Research Ethics Committee (Medical) (M140251) and by the Stellenbosch University Health Research Ethics Committee (N09/11/282). Parents/legal caregivers gave written informed consent for participation in the study, and assent was obtained from children older than 7 years of age who showed adequate understanding.

## RESULTS

**FIG 2:**
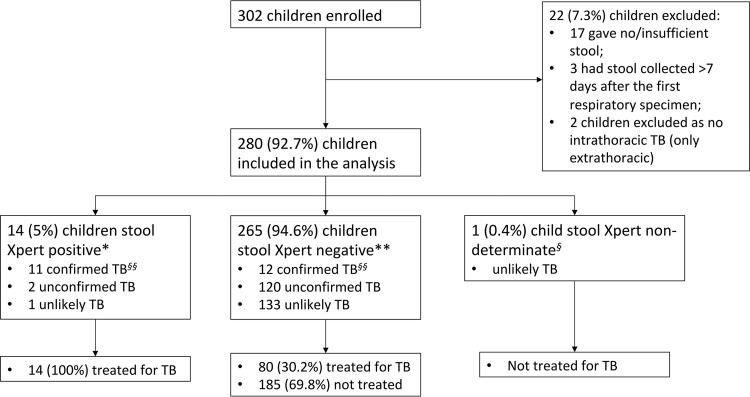
STARD cohort flow diagram, illustrating stool culture results by consensus case definition (13).

From December 2014 to September 2015, 302 children were enrolled; 280 (92.7%) were included in the final analysis (Fig. 2).

The cohorts from sites 1 and 2 differed significantly in clinical presentation and certainty of TB disease (Table 1). Children from site 1 had a higher prevalence of severe malnutrition, perinatal HIV exposure, and HIV infection but significantly lower prevalence of TB exposure and TB infection and of radiological findings considered typical of PTB. At site 2, all but one child had ≥3 respiratory specimens collected for Xpert and TB culture, whereas >70% of children from site 1 only had one respiratory specimen collected. Consequently, a significantly higher proportion of confirmed TB was detected at site 2 (20/132; 15.2%) than at site 1 (3/148; 2.0%).

**TABLE 1:** Demographic, clinical, and bacteriological characteristics of the two study cohorts

Characteristic^*a*^	Value [no. (%) unless otherwise indicated] for:			*P* value
	All children (*n* = 280)	Children at site:		
		1 (*n* = 148)	2 (*n* = 132)	
Median mo of age (IQR)	15.5 (10.6–29.1)	15.5 (10.9–24.3)	16.6 (5.2–34.3)	0.856
Male	158 (56.4)	86 (58.1)	72 (54.6)	0.548
Ethnicity				
Mixed race	85 (30.4)	15 (10.1)	70 (53.0)	<0.001
Black African	191 (68.1)	129 (87.2)	62 (47.0)	
Indian	3 (1.1)	3 (2.0)		
Caucasian	1 (0.4)	1 (0.7)		
Perinatal HIV exposure	96 (34.3)	63 (42.8)	33 (25.0)	0.009
HIV infected	35 (12.5)	24 (16.2)	11 (8.3)	0.049
On ART at presentation	23 (65.7)	16 (66.7)	7 (63.6)	0.861
Previous antituberculosis treatment	19 (6.8)	6 (4.1)	13 (9.8)	0.054
Median WAZ (IQR)	−2.3 (−3.7 to −1.1)	−3.2 (−4.2 to −2.2)	−1.4 (−2.2 to −0.6)	<0.001
Median HAZ (IQR) (*n* = 277)	−1.8 (−2.9 to −0.9)	−1.8 (−3.2 to −0.9)	−1.8 (−2.8 to −0.9)	0.316
WAZ of <−2	160 (57.1)	117 (79.1)	43 (32.6)	<0.001
Evidence of BCG immunization	267 (95.4)	142 (95.9)	125 (94.7)	0.062
≥1 well-defined TB symptom	206 (73.6)	111 (75.0)	95 (72.0)	0.566
TST positive	27 (17.7) (*n* = 152)	2 (4.0) (*n* = 50)	25 (24.5) (*n* = 102)	0.002
Exposure to identified TB source case	100 (35.7)	17 (11.5)	73 (55.3)	<0.001
CXR typical of TB (%)	33 (12.8) (*n* = 258)	8 (6.2) (*n* = 130)	25 (19.5) (*n* = 128)	0.001
Investigated by Xpert/culture with:				
1 respiratory specimen	105 (37.5)	105 (70.9)	0	
2 respiratory specimens	44 (15.7)	43 (29.1)	1 (0.8)	
≥3 respiratory specimens	131 (46.8)	0	131 (99.2)	<0.001
Treated for TB^*b*^ (%)	94 (33.6)	42 (28.4)	52 (39.4)	0.067
Clinical case categories				
Confirmed TB^*c*^	23 (8.2)	3 (2.0)	20 (15.2)	<0.001
Smear positive	4 (17.4)	1 (33.3)	3 (15.0)	
Xpert positive	16 (69.6)	3 (100)	13 (65.0)	
Culture positive	22 (95.7)	2 (66.6)	20 (100)	
Unconfirmed TB^*d*^	122 (43.6)	60 (40.5)	62 (47.0)	
Unlikely TB	135 (48.2)	85 (57.4)	50 (37.9)	
Follow-up status at mo 2				
Attended follow-up	259 (92.5)	128 (86.5)	131 (99.2)	<0.001
Lost to follow-up	15 (5.4)	14 (9.5)	1 (0.8)	0.001
Died	6 (2.1)	6 (4.1)	0	0.02

a IQR, interquartile range; ART, antiretroviral treatment; WAZ, weight-for-age Z score according to UK growth charts of 1990 (23); BCG, Mycobacterium bovis bacillus Calmette-Guérin; TST, Mantoux tuberculin skin test; CXR, chest radiograph.

b Includes children initiated on antituberculosis treatment within 2 months of enrollment.

c Includes only children confirmed by respiratory specimens. All smear-positive cases were also Xpert and culture positive; 15/16 Xpert-positive cases were also culture positive; 15/22 culture-positive cases were also Xpert positive.

d Three children with unconfirmed TB (i.e., mycobacteriology negative on respiratory specimens) were stool Xpert positive, all from site 1.

A clinical decision was made to start antituberculosis treatment in 88 (31.4%) children at the time of enrollment and in another 6 (2.1%) during the 8 weeks following enrollment. Considering the results for all respiratory specimens collected and tested by Xpert and culture (but excluding stool Xpert as the index test under evaluation, following STARD guidelines [14]) and all available clinical data, 23 (8.2%) children overall had confirmed TB (15 by Xpert and culture, 7 only by culture, and 1 only by Xpert), 122 (43.6%) were unconfirmed for TB, and 135 (48.2%) were unlikely to have TB using international consensus definitions for diagnostic studies (13).

Follow-up was completed in 259 (92.5%) children: 15 (5.4%) were lost to follow-up and 6 (2.1%) (all from site 1) died. The deaths were from infectious causes complicated by multiple comorbid conditions (HIV, malnutrition, and cerebral palsy). Two deaths were due to TB, one with multidrug resistance.

### Diagnostic performance of one stool specimen.

Stools from 268/279 (96.1%) and 267/279 (95.7%) children tested by Xpert-06 (Xpert on 0.6 g stool) and Xpert-S (Xpert on stool swab), respectively, yielded final determinate results and were included in the analyses (see Fig. S1a and b in the supplemental material).

Case detection from stool and respiratory specimens at site 1 was low: only 3 children had confirmed TB from respiratory specimens, 2 (66.7%) of whom were also stool Xpert positive. Three additional children were stool Xpert positive but negative on all respiratory specimens. In addition, site 1 had no positive results from Xpert-S. At site 2, 20 children were microbiologically confirmed on respiratory specimens, of whom 9 (45.0%) were also stool Xpert positive. All cases detected on stool had positive results on respiratory specimens. Given the small numbers detected at site 1, diagnostic accuracy analyses from the two sites were combined. Individual site analyses are presented in Tables S1 and S2.

Compared to Xpert on the first respiratory specimen, the two stool testing methods performed similarly, with sensitivity of 44.4% (4/9; 95% CI, 13.7 to 78.8) and specificity of >99% (Table 2). Three children had stool specimens positive on Xpert-06 and Xpert-S, while two were positive only on Xpert-06 and one only on Xpert-S. Xpert-06 detected M. tuberculosis in two children with negative respiratory Xpert, while Xpert-S detected one additional child with negative respiratory Xpert.

**TABLE 2:** Diagnostic value of a single stool specimen tested by 0.6-g- and swab-sample protocols (per-participant analysis)^*a*^

Stool Xpert protocol	Parameter	Ratio (%; 95% CI) using indicated assay on 1st respiratory specimen as reference standard	
		Xpert	Culture
0.6 g		*n* = 259^*b*^	*n* = 240^*c*^
	Sensitivity	4/9 (44.4; 13.7–78.8)	4/16 (25.0; 7.3–52.4)
	Specificity	248/250 (99.2; 97.1–99.9)	222/224 (99.1; 96.8–99.9)
	PPV	4/6 (66.7; 22.3–95.7)	4/6 (66.7; 22.3–95.7)
	NPV	248/253 (98.0; 95.4–99.4)	222/234 (94.9; 91.2–97.3)
Swab		*n* = 259^*d*^	*n* = 236^*e*^
	Sensitivity	4/9 (44.4; 13.7–78.8)	4/16 (25.0; 7.3–52.4)
	Specificity	249/250 (99.6; 97.8–100)	219/220 (99.5; 97.5–100)
	PPV	4/5 (80.0; 28.4–99.5)	4/5 (80.0; 28.4–99.5)
	NPV	249/254 (98.0; 95.5–99.4)	219/231 (94.8; 91.1–97.3)

a PPV, positive predictive value; NPV, negative predictive value; CI, confidence interval. The ratios are as follows: for sensitivity, number positive by Xpert/number positive by reference assay; for specificity, number negative by Xpert/number negative by reference assay; PPV, number positive by Xpert which were also positive by reference assay/total number positive by Xpert; NPV, number negative by Xpert which were also negative by reference assay/total number negative by Xpert.

b One child with only the swab method on stool 1, 11 children with nondeterminate stool Xpert results, and 9 with nondeterminate respiratory Xpert results were excluded.

c One child with only the swab method on stool 1, 11 children with nondeterminate stool Xpert results, 2 with no respiratory culture done, and 26 with contaminated or lost respiratory cultures were excluded.

d One child with only the 0.6-g method on stool 1, 12 children with nondeterminate stool Xpert results, and 8 with nondeterminate respiratory Xpert results were excluded.

e One child with only the 0.6-g method on stool 1, 12 children with nondeterminate stool Xpert results, 2 with no respiratory culture done, and 29 with contaminated or lost respiratory cultures were excluded.

The sensitivity compared to the results for one respiratory specimen culture was lower for both methods, with sensitivity of 25.0% (4/16; 95% CI, 7.3 to 52.4) and specificity remaining >99% (Table 2). Although Xpert-06 and Xpert-S still added two and one confirmed diagnoses, respectively, respiratory culture detected 12 children who were negative on stool Xpert.

### Incremental value of a second stool test.

A second stool test was done in 249 (88.9%) children (Fig. S2a and b): in 132 (53.0%), a separate stool specimen was collected, while in 117 (47.0%), the first stool specimen was retested.

For Xpert-06, a second stool test gave valid results in 8/11 (72.7%) specimens that were nondeterminate on the first testing (after repeat testing of stool 1 nondeterminates). Therefore, the nondeterminate rate for stool 1 and stool 2 combined was 3/279 (1.1%; 3 samples did not have a second test). The second stool test added 5 additional confirmed cases (*n* = 9 total cases detected from second stool specimen) to the 6 cases which had already been detected by stool specimen 1 (*n* = 11 total cases positive on Xpert-06; incremental detection of 83.3%).

For Xpert-S, a second stool test gave valid results in 11/12 (91.7%) specimens that were nondeterminate on the first testing. Therefore, the nondeterminate rate for stool 1 and stool 2 combined was 1/280 (0.4%; one sample did not have a second test). A second stool test added two additional confirmed cases (*n* = 5 total cases detected from stool 2), to the 5 cases already detected by stool 1 (*n* = 7 total cases positive on Xpert-S; incremental detection of 40%).

The combined sensitivity of two stool tests versus the first respiratory Xpert increased to 70.0% (95% CI, 34.8 to 93.3) for Xpert-06 and to 50.0% (95% CI, 18.7 to 81.3) for Xpert-S while retaining high specificity for both methods (Table 3). The slightly lower specificity observed for Xpert-06 was due to three cases who were positive on stool but negative on respiratory Xpert. Compared to the first respiratory culture, the sensitivity was 41.2% (95% CI, 18.4 to 67.1) for Xpert-06 and 35.3% (95% CI, 14.2 to 61.7) for Xpert-S, with a specificity of >99% for both methods (Table 3).

**TABLE 3:** Combined diagnostic value of stools 1 and 2^*a*^

Stool Xpert protocol	Parameter	Ratio (%; 95% CI) using indicated assay on 1st respiratory specimen as reference standard	
		Xpert	Culture
0.6 g		*n* = 267^*b*^	*n* = 244^*c*^
	Sensitivity	7/10 (70.0; 34.8–93.3)	7/17 (41.2; 18.4–67.1)
	Specificity	253/257 (98.4; 96.1–99.6)	223/227 (98.2; 95.5–99.5)
	PPV	7/11 (63.6; 30.8–89.1)	7/11 (63.6; 30.8–89.1)
	NPV	253/256 (98.8; 96.6–99.8)	223/233 (95.7; 92.2–97.9)
Swab		*n* = 270^*d*^	*n* = 247^*e*^
	Sensitivity	5/10 (50.0; 18.7–81.3)	6/17 (35.3; 14.2–61.7)
	Specificity	258/260 (99.2; 97.2–99.9)	229/230 (99.6; 97.6–100)
	PPV	5/7 (71.4; 29.0–96.3)	6/7 (85.7; 42.1–99.6)
	NPV	258/263 (98.1; 95.6–99.4)	229/240 (95.2; 91.6–97.6)

a PPV, positive predictive value; NPV, negative predictive value; CI, confidence interval. The ratios are as follows: for sensitivity, number positive by stool 1 or 2 Xpert/number positive by reference assay; for specificity, number negative by stool 1 and 2 Xpert/number negative by reference assay; PPV, number positive by stool 1 or 2 Xpert which were also positive by reference assay/total number positive by stool 1 or 2 Xpert; NPV, number negative by stool 1 and 2 Xpert which were also negative by reference assay/total no. negative by stool 1 and 2 Xpert.

b One child with only swab method on stool 1, 3 children with nondeterminate stool Xpert results and 9 with nondeterminate respiratory Xpert results were excluded.

c One child with only swab method for stool 1, 3 children with nondeterminate stool Xpert results, 3 with no respiratory culture done, and 29 with contaminated or lost respiratory cultures were excluded.

d One child with nondeterminate stool Xpert results and 9 with nondeterminate respiratory Xpert results were excluded.

e One child with nondeterminate stool Xpert results, 3 with no respiratory culture done, and 29 with contaminated or lost respiratory cultures were excluded.

Comparing the clinical case definitions (13) to the combined results from stools 1 and 2, Xpert-06 was positive in 8/23 (34.8%) children with confirmed TB, 2/122 (1.6%) with unconfirmed TB, and 1/135 (0.7%) with unlikely TB. Xpert-S was positive in 7/23 (30.4%) children with confirmed TB and did not detect any unconfirmed or unlikely TB cases. Considering any positive stool Xpert result (from Xpert-06 or Xpert-S), 14 children were detected by testing on stool (Fig. 2). Three of the 14 children detected on stool Xpert had positive stool Xpert-06 but negative respiratory tests: two only had one IS collected, which was negative on smear, Xpert, and culture; one child had an IS, which was negative on all TB tests, and a GA, which was smear and Xpert negative, and the culture was contaminated. All three children were under 20 months of age and had no prior TB history. One was HIV infected with an acute presentation. The child had started antiretroviral therapy 2 weeks before enrollment and was critically ill with multiorgan dysfunction, multilobar pneumonia, and confirmed nosocomial sepsis. He had no known TB exposure. The child died before follow-up was completed. The other two were HIV negative, with symptoms and chest radiographs (CXR) suggestive of TB and with a good clinical response to antituberculosis treatment at the 8-week follow-up.

Using confirmed TB as the reference standard, the sensitivity of testing a single stool by both Xpert-06 and Xpert-S was 7/23 (30.4%; 95% CI 13.2 to 52.9%), versus 17/23 (73.9%; 95% CI 51.6 to 89.8%) for one respiratory culture. Using clinical TB (decision to treat for TB) as the reference, a single stool tested by Xpert-06 and Xpert-S had a sensitivity of 8/94 (8.51%; 95% CI 3.75 to 16.1%), versus 17/94 (18.1%; 95% CI 10.9 to 27.4%) for one respiratory culture.

All stool Xpert results reported low or very low semiquantitative values: of 15 positive Xpert-06 results (from 11 children), 6 (40.0%) were low and 9 (60.0%) very low; of 10 positive Xpert-S results (from 7 children), 7 (70%) were low and 3 (30%) very low.

Stool Xpert did not detect any rifampin resistance. In three of five children with rifampin resistance detected in respiratory specimens, the stool Xpert results were negative. In the other two children, Xpert-06 was rifampin indeterminate due to low bacillary loads and prolonged cycle threshold values (Table S3). Overall, 6/25 (24%) Xpert-positive stool specimens (from 5 children) gave indeterminate rifampin resistance results: 5/15 (33.3%) and 1/10 (10%) on Xpert-06 and Xpert-S, respectively. All six indeterminate results had “very low” semiquantitative values (Table S4). By comparison, none of the Xpert-positive respiratory specimens had indeterminate rifampin resistance.

### Factors associated with stool Xpert positivity.

On univariate analysis, factors associated with stool Xpert positivity were radiologically severe TB (*P* < 0.001), female sex (*P* = 0.03), and positive sputum smear status (*P* < 0.001). On multivariable analysis, only radiologically severe TB remained strongly associated with stool Xpert positivity. Smear status could not be included in the model as it predicted stool Xpert positivity perfectly (Table 4).

**TABLE 4:** Regression analysis exploring factors associated with stool Xpert positivity^*a*^

Variable	OR^*b*^	95% CI^*c*^	*P* value	aOR^*d*^	95% CI	*P* value
Sex						
Male	Reference					
Female	3.4	1.1–11.2	0.03	3.0	0.8–10.7	0.09
Age in mo	1.0	0.99–1.0	0.30			
HIV status						
Negative	Reference					
Positive	1.2	0.3–5.5	0.84			
Stool consistency						
Liquid	Reference					
Not liquid	0.90	0.5–1.6	0.73			
Stool collection time in relation to respiratory specimen collection						
After	Reference					
Same day/before	0.9	0.3–2.6	0.80			
Stool collection time in relation to TB treatment initiation						
After	Reference					
Same day/before	2.3	0.7–7.4	0.15			
TB disease severity						
Not severe	Reference					
Severe	22.1	6.5–75.4	<0.001	20.9	6.0–72.0	<0.001

a Any stool Xpert test positive; per-participant analysis.

b OR, odds ratio. “Reference” refers to the base or reference category used for the regression analyses.

c CI, confidence interval.

d aOR, adjusted odds ratio.

Although stool consistency was not associated with Xpert positivity, no positive stool Xpert results were obtained from liquid stools and only two were from solid stools (Table S5). None of the 14 stools with visible mucus were Xpert positive. One of four bloody stools was Xpert positive.

## DISCUSSION

This is the first large study, since initial proof-of-concept (10), to evaluate the performance of a novel centrifugation-free processing method for stool specimens, to assess its use with the Xpert MTB/RIF assay on stool to diagnose TB in children. Compared to microbiological confirmation using respiratory specimens, this method demonstrated diagnostic accuracy similar to those of recently published studies in young children (7, 8, 15), while studies enrolling mainly older children reported higher sensitivities for stool Xpert (9, 16, 17). This is most likely due to the lower bacillary concentrations present in respiratory specimens and, hence, in stools of young children, who seldom present with adult-type (cavitating) TB (18, 19). We have also previously found that a more severe spectrum of TB disease as evaluated by CXR (associated with higher mycobacterial load) was strongly predictive of stool Xpert positivity using a different stool processing protocol (7) in children enrolled in the parent study from Cape Town, independently of age.

The aim of this study was to develop and evaluate a simple method to process stool, a noninvasive specimen, for use with the Xpert assay in children. This processing method is better suited to underresourced settings, as it does not require centrifugation. We showed that a simple swab gave results similar to those of the 0.6-g-sample method, although due to the small number of positive results, the comparison was not adequately powered to show equivalence. Stool swabs have previously been used for Xpert testing; however, a centrifugation-dependent method was used, which also required the stool mass collected on the swab to be weighed (8). Our method is more feasible for clinical and laboratory settings with minimal infrastructure, and as this study illustrates, the process could be performed at a point-of-care site situated close to the patient's ward. Conversely, although not statistically significant, the higher initial stool sample volume (0.6 g) did result in the detection of M. tuberculosis in seven additional children compared to swab samples, which added three diagnoses (Fig. 2), indicating that further improvements using a larger stool volume and multiple samplings could improve sensitivity.

Our study also shows that the addition of a second stool test, either from a separate stool specimen or from retesting the same stool, had a substantial benefit for the diagnostic yield and for the rate of nondeterminate results. Of the initial nondeterminate results, none were due to pressure aborts, and 2 of the error results were instrument related. The remaining nondeterminate results are likely to have been caused by inhibition. Given the success of repeat testing using residual raw stool, it is unlikely that specimen processing could have been the cause of the nondeterminate results. In this study, whether a second stool was tested or the first stool was retested, the initial starting material was raw stool (not stool stored in buffer). The first step of the stool processing protocol involved adding the stool processing buffer and gently vortexing to homogenize the sample (Fig. 1). This protocol, including standing times, was followed in a standard way for all specimens. The improvement in nondeterminate results with second testing is probably explained by the inhomogeneous property of stool, resulting in PCR inhibitors and particulate matter not being completely homogenized with rapid vortexing. Experience from the Rutgers laboratory confirms that discrepant results from repeat testing of the same stool specimens frequently occurs. It is also important to note that initial valid stool results were not repeated—it is possible that if all stool specimens were tested twice, a similar proportion of second tests would have yielded nondeterminate results as for the first tests. The incremental yield of a second stool test is likely explained by the paucibacillary nature of pediatric tuberculosis, where, as also with respiratory specimens, increasing the number of specimens/tests increases diagnostic yield (10). Although additional testing results in higher costs, restricting additional tests to nondeterminate or negative Xpert results should be considered in cases with high pretest probability of disease or where confirmation of TB is most important, such as in infants and HIV-infected children or those with exposure to a drug-resistant source case.

We observed a high proportion of indeterminate rifampin results in stools, in line with very low Xpert semiquantification. It is known that in paucibacillary specimens, the rifampin resistance results in G4 Xpert cartridges may not be reliable (20, 21). Although this could potentially limit the utility of Xpert to detect rifampin-resistant TB from stool, a more sensitive assay, such as the Xpert ultra, may be able to overcome this limitation (22).

Ours is the first study to explore stool consistency in relation to diagnostic yield by the Xpert assay. Although detection of M. tuberculosis was not associated with stool consistency, it is interesting to note that no liquid stools generated a positive Xpert result. Of 46 liquid stool specimens, 42 were collected in ordinary diapers (2 of the remainder were collected in urine bags and 2 in the potty), which may have resulted in a large part of the stool being soaked up into the diaper. Other explanations may include inhibitors in diarrheal stools, as well as higher dilution with lower concentration of M. tuberculosis DNA, or possibly, none of the children with liquid stools had active TB.

A limitation of our study was the enrollment of two substantially different cohorts of children. At site 1, TB testing was part of a package of investigations for very ill children with a high burden of comorbid diseases, and Xpert testing was performed onsite directly on the raw sputum specimen. Site 2 had more strictly defined entry criteria, the pretest probability of TB disease was higher, and the Xpert test was performed in a laboratory setting on the concentrated pellet. In addition, site 2 collected more respiratory specimens for TB investigation, which resulted in a higher proportion of children confirmed by both respiratory specimens and stool. We attempted to address these differences by comparing stool Xpert results to a reference standard that was common to both groups: a single respiratory specimen tested by Xpert and culture. However, overall, site 2 contributed the majority of positive test results and drove the results for the sensitivity analyses. The small number of confirmed cases in this study overall also resulted in wide confidence intervals around all the estimates.

Three children from site 1 had positive stool Xpert but negative respiratory mycobacteriology. Although in at least two of the three children, the stool Xpert results were likely true positives, it remains important to optimize the reference standard in order to adequately evaluate new diagnostic tests for pediatric TB. In children, collecting a minimum of two high-quality respiratory specimens, ensuring appropriate specimen storage and transport, and optimizing laboratory processes (such as specimen concentration before testing) are critical.

Our results demonstrate the superior value of respiratory specimens for the diagnosis of intrathoracic TB in children: a single respiratory culture detected more than double (*n* = 17) the number of children detected by a single stool (*n* = 8). These results, therefore, support efforts to promote and strengthen the capacity for collection and testing of respiratory specimens in children for microbiological investigation of TB, as stool testing remains inadequately sensitive and largely limited to the detection of severe forms of TB. In settings where the use of empirical treatment based on clinical algorithms is high, stool-based diagnosis has limited value. However, in settings where children with TB present with advanced disease and where confirmation is required to access treatment but resources are scarce, the use of stools may improve case detection.

### Conclusions.

Despite the encouraging performance of our simple, centrifugation-free stool-processing method and the value shown in testing a second stool specimen, our study reinforces that stools cannot yet replace respiratory specimens for detection of M. tuberculosis in children. Children with nonsevere PTB are less likely to be detected with stool Xpert, limiting the utility of this diagnostic modality primarily to children with severe disease. The diagnostic yield of a single respiratory culture was considerably superior to that of stool Xpert, allowing for completion of full drug susceptibility testing (DST). Culture of respiratory specimens remains the most sensitive diagnostic strategy for pediatric TB if resources are available. A major benefit of Xpert, however, remains the rapid turnaround time and ability to screen for rifampin resistance. In settings where children present with severe disease and where the capacity for respiratory specimen collection is limited, more sensitive rapid assays, such as Xpert ultra, combined with an easy-to-use SP kit could prove even more useful and should be urgently evaluated.

## Supplementary Material

Supplemental file 1
