# Supplementary material for: Molecular Detection of Mycobacterium tuberculosis from Stools in Young Children by Use of a Novel Centrifugation-Free Processing Method
Source: J Clin Microbiol. 2018 Aug 27;56(9):e00781-18. doi: 10.1128/JCM.00781-18 (PMC6113478; doi:10.1128/JCM.00781-18)
Supplement: Supplemental file 1 [file zjm999096098s1.pdf]

Figure S1a. Flow diagram showing stool 1 specimens (1 stool per participant) finally included in the Xpert-06 analysis

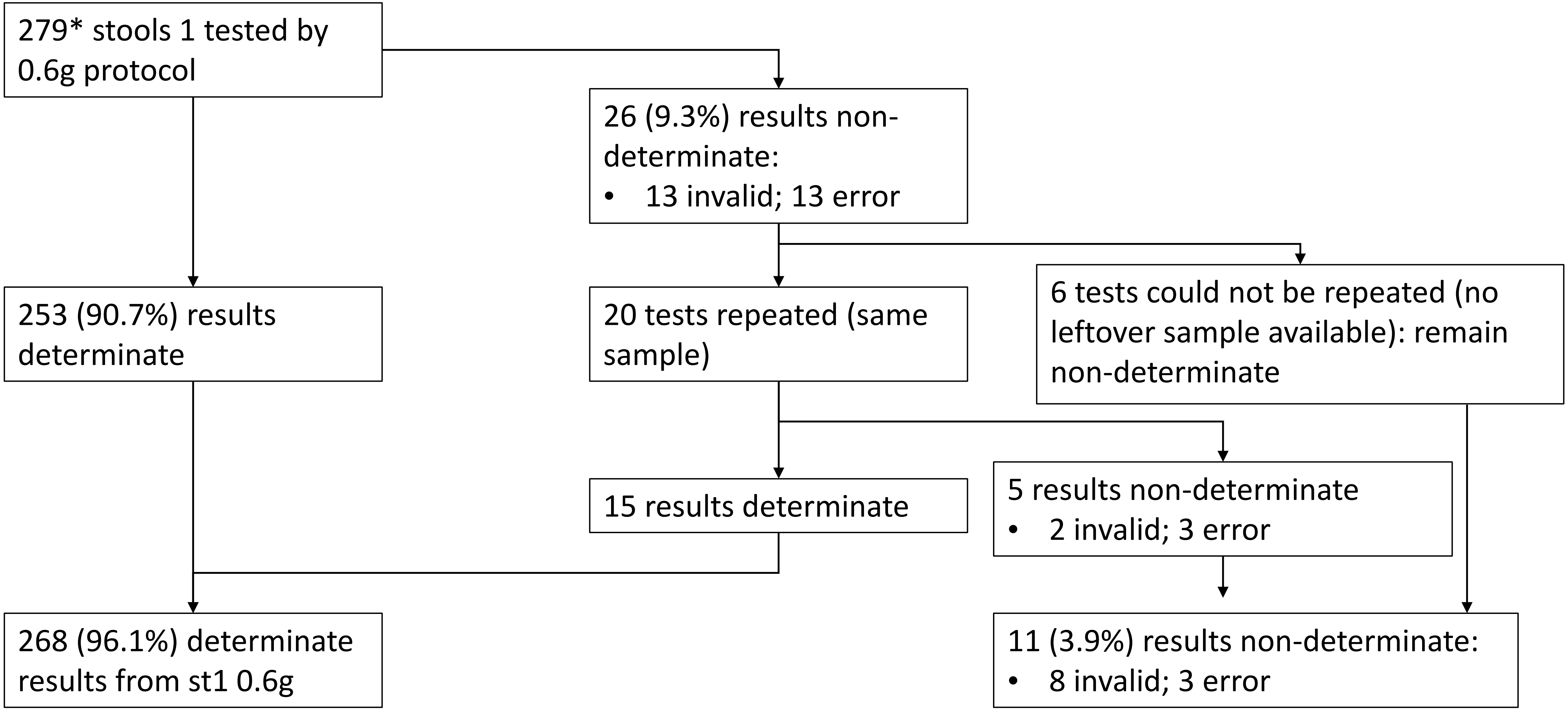

*Stool from one child was only tested by swab

Figure S1b. Flow diagram showing stool 1 specimens (1 stool per participant) finally included in the Xpert-S analysis

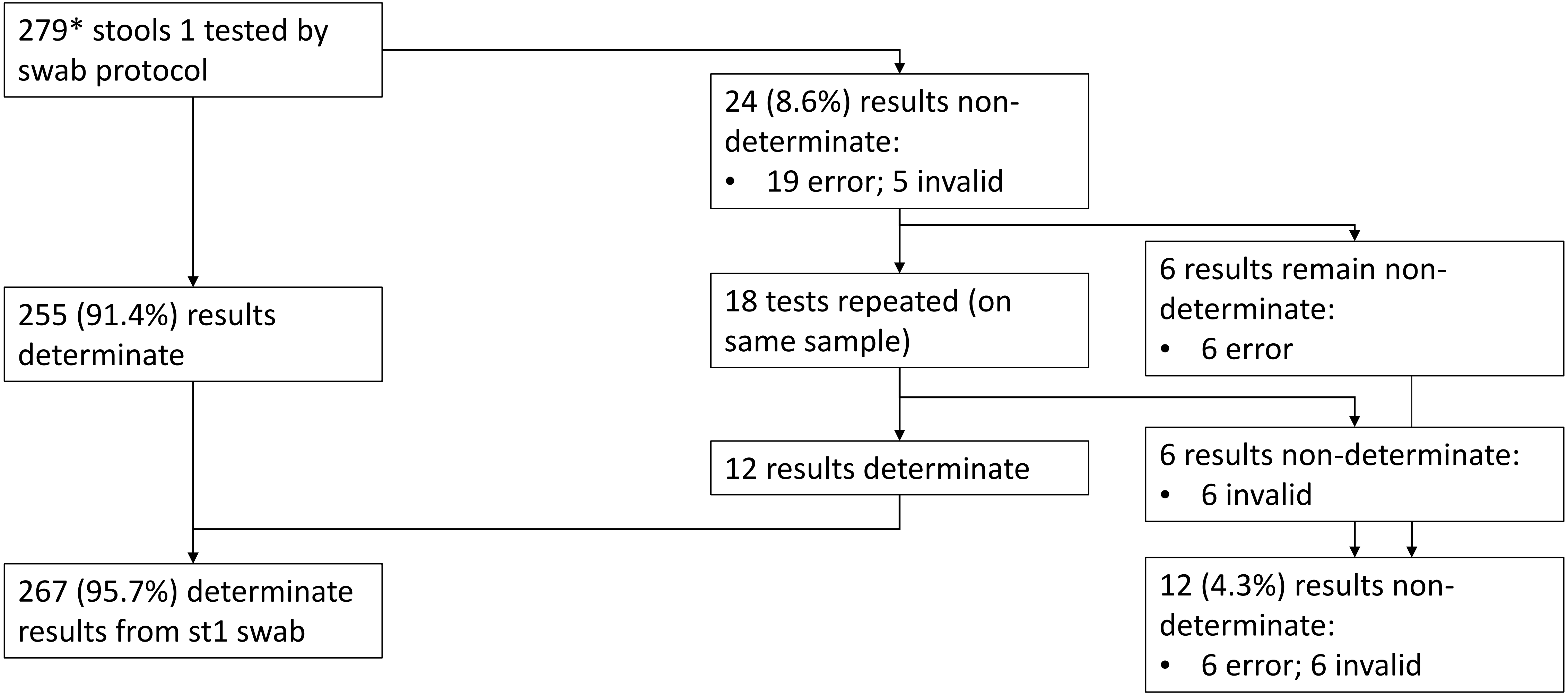

*Stool from one child was only tested by 0.6g protocol

Figure S2a. Flow diagram showing stool 2 specimens (1 stool per participant) finally included in the Xpert-06 analysis

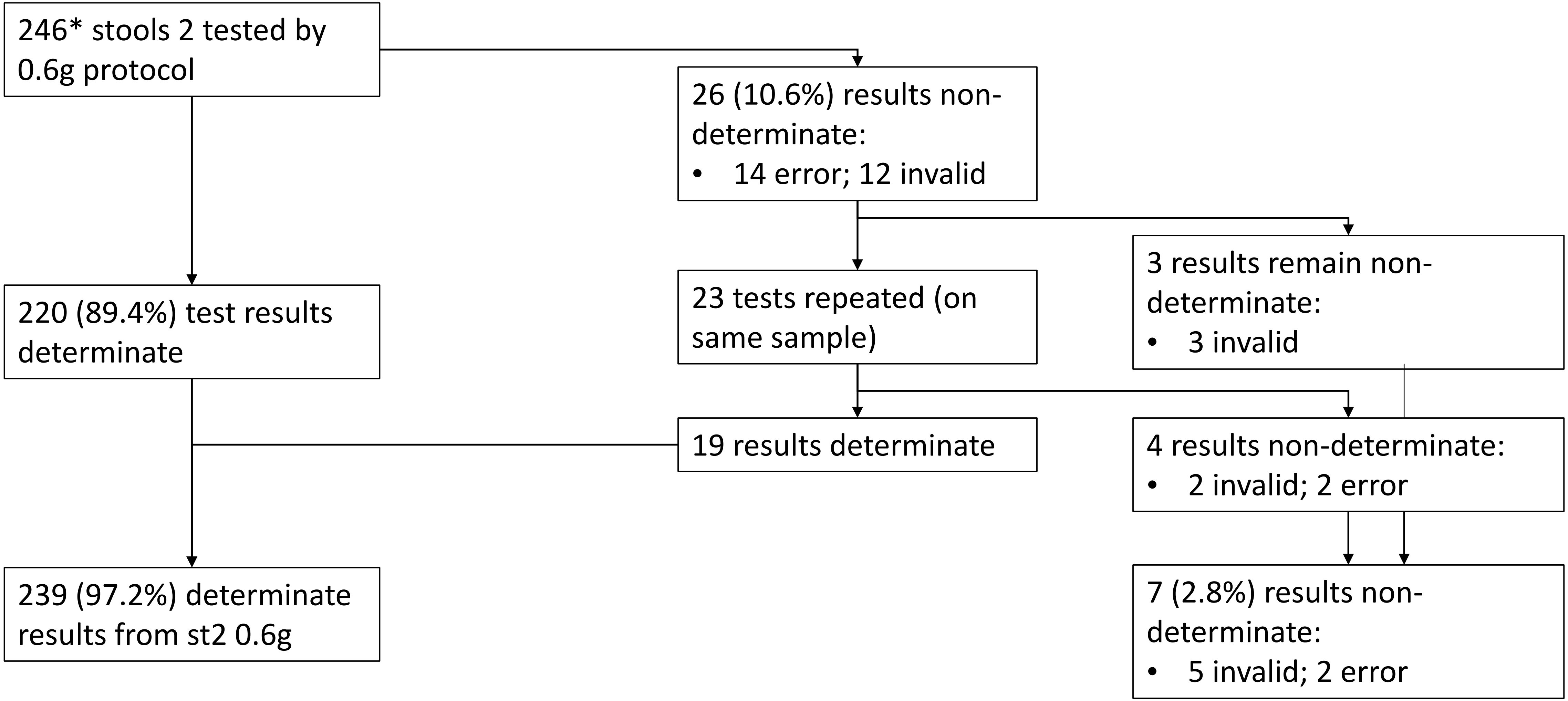

*249 collected; 1 only tested by swab and 2 collected >7 days after first respiratory specimen so excluded

Figure S2b. Flow diagram showing stool 2 specimens (1 stool per child) finally included in the Xpert-S analysis

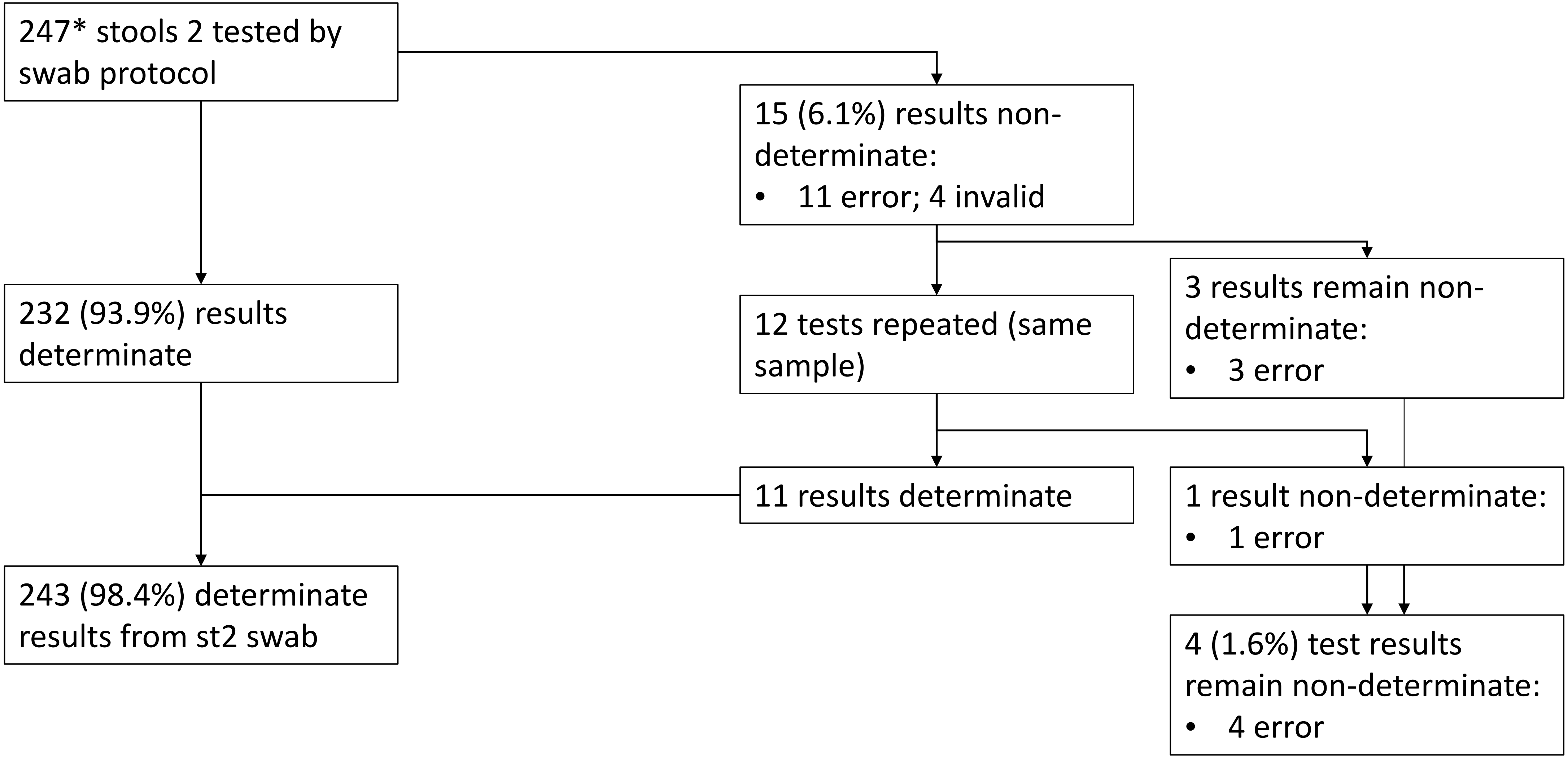

*249 collected; 2 collected >7 days after first respiratory specimen so excluded

Table S1. The diagnostic value of a single stool specimen tested by 0.6g and swab protocol (per participant analysis)

Stool Xpert method	Reference standard					
	Xpert on 1st respiratory specimen			Culture on 1st respiratory specimen		
0.6g	Site 1 [n=130]	Site 2 [n=129]	Both sites [n=259]*	Site 1 [n=120]	Site 2 [n=120]	Both sites [n=240]**
Sensitivity (%; 95% CI)	1/1; (100; 2.5-100.0)	3/8; (37.5; 8.5-75.5)	4/9; (44.4; 13.7-78.8)	1/1; (100; 2.5-100)	3/15; (20; 4.3-48.1)	4/16; (25.0; 7.3- 52.4)
Specificity (%; 95% CI)	127/129; (98.4; 94.5-99.8)	121/121; (100; 97.0-100)	248/250; (99.2; 97.1- 99.9)	117/119; (98.3; 94.1-99.8)	105/105; (100; 96.5-100)	222/224; (99.1; 96.8- 99.9)
PPV (%; 95% CI)	1/3; (33.3; 0.84-90.6)	3/3; (100; 29.2-100)	4/6; (66.7; 22.3- 95.7)	1/3; (33.3; 0.84-90.6)	3/3; (100; 29.2-100)	4/6; (66.7; 22.3- 95.7)
NPV (%; 95% CI)	127/127; (100; 97.1-100)	121/126; (96.0; 91.0-98.7)	248/253; (98.0; 95.4- 99.4)	117/117; (100; 96.9-100)	105/117; (89.8; 82.8-94.6)	222/234; (94.9; 91.2-97.3)
Swab	Site 1 [n=131]	Site 2 [n=128]	Both sites [n=259][§]	Site 1 [n=117]	Site 2 [n=119]	Both sites [n=236][§§]
Sensitivity (%; 95% CI)	0/1; (0; 0.0-97.5)	4/8; (50.0; 15.7-84.3)	4/9; (44.4; 13.7-78.8)	0/1; (0; 0-97.5)	4/15; (26.7; 7.8-55.1)	4/16; (25.0; 7.3- 52.4)
Specificity (%; 95% CI)	130/130; (100; 97.2-100)	119/120; (99.2; 95.4-100)	249/250; (99.6; 97.8- 100)	116/116; (100; 96.9-100)	103/104; (99.0; 94.8-100)	219/220; (99.5; 97.5- 100)
PPV (%; 95% CI)	-	4/5; (80.0; 28.4-99.5)	4/5; (80.0; 28.4- 99.5)	-	4/5; (80.0; 28.4-99.5)	4/5; (80.0; 28.4-99.5)
NPV (%; 95% CI)	130/131; (99.2; 95.8-100)	119/123; (96.7; 91.9-99.1)	249/254; (98.0; 95.5-99.4)	116/117; (99.1; 95.3-100)	103/114; (90.4; 83.4-95.1)	219/231; (94.8; 91.1-97.3)

*PPV: positive predictive value; CI: confidence interval; NPV: negative predictive value; *1 child with only swab method on stool 1; 11 children with non-determinate stool Xpert results and 9 with non-determinate respiratory Xpert results were excluded; **1 child with only swab method on stool 1; 11 children with non-determinate stool Xpert result; 2 with no respiratory culture done and 26 with contaminated or lost respiratory cultures were excluded; [§]1 child with only 0.6g method on stool 1; 12 children with non-determinate stool Xpert results and 8 with non-determinate respiratory Xpert results were excluded; [§§]1 child with only 0.6g method on stool 1; 12 children with non-determinate stool Xpert results, 2 with no respiratory culture done and 29 with contaminated or lost respiratory cultures were excluded.*

Table S2. The combined diagnostic value of stool 1 and 2

Stool Xpert method	**Reference standard**					
	Xpert on 1st respiratory specimen			Culture on 1st respiratory specimen		
0.6g	Site 1 [n=137]	Site 2 [n=130]	Both sites [n=267]*	Site 1 [n=123]	Site 2 [n=121]	Both sites[n=244]**
Sensitivity (%; 95% CI)	1/1; (100; 2.5-100.0)	6/9; (66.7; 29.9-92.5)	7/10; (70.0; 34.8-93.3)	1/1; (100; 2.5-100.0)	6/16; (37.5; 15.2-64.6)	7/17; (41.2; 18.4- 67.1)
Specificity (%; 95% CI)	132/136; (97.1; 92.6-99.2)	121/121; (100; 97.0-100)	253/257; (98.4; 96.1- 99.6)	118/122; (96.7; 91.8-99.1)	105/105; (100; 96.5-100)	223/227; (98.2; 95.5- 99.5)
PPV (%; 95% CI)	1/5; (20.0; 0.5-71.6)	6/6; (100; 54.1-100)	7/11; (63.6; 30.8-89.1)	1/5; (20.0; 0.5-71.6)	6/6; (100; 54.1-100)	7/11; (63.6; 30.8-89.1)
NPV (%; 95% CI)	132/132; (100; 97.2-100)	121/124; (97.6; 93.1-99.5)	253/256; (98.8; 96.6-99.8)	118/118; (100; 96.9-100)	105/115; (91.3; 84.6-85.8)	223/233; (95.7; 92.2-97.9)
Swab	Site 1 [n=139]	Site 2 [n=131]	Both sites [n=270] §	Site 1 [n=125]	Site 2 [n=128]	Both sites [n=247] §§
Sensitivity (%; 95% CI)	0/1; (0; 0.0-97.5)	5/9; (55.6; 21.2-86.3)	5/10; (50.0; 18.7-81.3)	0/1; (0; 0.0-97.5)	6/16; (37.5; 15.2-64.6)	6/17; (35.3; 14.2- 61.7)
Specificity (%; 95% CI)	138/138; (100; 97.4-100)	120/122; (98.4; 94.2-99.8)	258/260; (99.2; 97.2-99.9)	124/124; (100; 97.1-100)	105/106; (99.1; 94.9-100)	229/230; (99.6; 97.6- 100)
PPV (%; 95% CI)	-	5/7; (71.4; 29.0-96.3)	5/7; (71.4; 29.0-96.3)	-	6/7; (85.7; 42.1-99.6)	6/7; (85.7; 42.1-99.6)
NPV (%; 95% CI)	138/139; (99.3; 96.1-100)	120/124; (96.8; 91.9-99.1)	258/263; (98.1; 95.6-99.4)	124/125; (99.2; 95.6-100)	105/115; (91.3; 84.6-95.8)	229/240; (95.2; 91.6-97.6)

*PPV: positive predictive value; CI: confidence interval; NPV: negative predictive value; *1 child with only swab method on stool 1; 3 children with non-determinate stool Xpert results and 9 with non-determinate respiratory Xpert results were excluded; **1 child with only swab method on stool 1; 3 children with non-determinate stool Xpert result; 3 with no respiratory culture done and 29 with contaminated or lost respiratory cultures were excluded; § 1 child with non-determinate stool Xpert results and 9 with non-determinate respiratory Xpert results were excluded; §§1 child with non-determinate stool Xpert results, 3 with no respiratory culture done and 29 with contaminated or lost respiratory cultures were excluded.*

Table S3. Cycle threshold (Ct) values for Xpert-positive stool results for the 2 children with rifampicin resistance detected on respiratory specimens who were also stool-positive, but with indeterminate rifampicin results

Sample ID	Test Result	SPC	Probe E	Probe D	Probe B	Probe C	Probe A	min rpoB Ct	deltaCt_max
096_ST1_0.6g	MTB DETECTED VERY LOW; Rif Resistance INDETERMINATE	24.7	0	32.5	32.2	33	33.2	32.2	-32.2
096_ST2_0.6g	MTB DETECTED VERY LOW; Rif Resistance INDETERMINATE	24.6	0	32.8	32.1	33.2	32.9	32.1	-32.1
391_ST2_0.6g	MTB DETECTED VERY LOW; Rif Resistance INDETERMINATE	22.5	37.6	35.4	34.8	35.3	35.7	34.8	2.8

ST1: stool specimen 1; ST2: stool specimen 2; SPC: sample processing control

Table S4. All stool results for the 14 children who had *M. tuberculosis complex* detected on stool Xpert.

PID	Final S1 Xp 06 res	Final S1 Xp 06 semiq	Final S1 Xp 06 rif res	Final S1 Xp Sw res	Final S1 Xp Sw semiq	Final S1 Xp Sw rif res	Final S2 Xp 06 res	Final S2 Xp 06 semiq	Final S2 Xp 06 rif res	Final S2 Xp Sw res	Final S2 Xp Sw semiq	Final S2 Xp Sw rif res	Summary results for respiratory specimens
51	POS	VL	ND	NEG	.	.	NEG			NEG			Only 1 resp specimen: neg by Xpert and culture
83	NEG			NEG			POS	VL	ND	NEG			Only 1 resp specimen: neg by Xpert and culture
96	POS	VL	IND	NEG			POS	VL	IND	NEG			Only 1 resp specimen: pos by Xpert (rif detected); pos by culture (rif and inh resistant on LPA)
145	NEG			I/E			POS	VL	IND	NEG			2 resp specimens: both neg by Xpert and culture
152	POS	VL	IND	NEG			NEG			NEG			2 resp specimens: one neg; one pos by Xpert (rif not detected) and pos by culture (rif and inh susceptible on LPA)
366	NEG			NEG			NEG			POS	VL	IND	4 resp specimens: one pos by Xpert (rif not detected); two pos by culture (rif and inh susceptible on LPA)
372	NEG			NEG			POS	VL	ND	NEG			4 resp specimens: 4 pos by Xpert (rif not detected); one pos by culture (rif and inh susceptible on LPA)
374	POS	VL	ND	POS	L	ND	POS	L	ND	POS	L	ND	4 resp specimens: all pos by Xpert (rif not detected) and by culture (rif and inh susceptible on LPA)
391	NEG			NEG			POS	VL	IND	NEG			4 resp specimens: all pos by Xpert (rif not detected in 3; rif detected and deemed false positive in 1) and by culture (rif and inh susceptible on LPA)
394	I/E			I/E			POS	L	ND	POS	L	ND	4 resp specimens: all pos by Xpert (rif not detected) and by culture (rif and inh susceptible on LPA)
458	POS	L	ND	POS	L	ND	POS	L	ND	POS	L	ND	3 resp specimens: all pos by Xpert (rif not detected) and by culture (rif and inh susceptible on LPA)
497	NEG			POS	VL	ND	NEG			NEG			4 resp specimens: one pos by Xpert (rif not detected) and pos by culture (rif and inh susceptible on LPA)
502	NEG			POS	VL	ND	NEG			NEG			4 resp specimens: two pos by Xpert (rif not detected) and pos by culture (rif and inh susceptible on LPA)
503	POS	L	ND	POS	L	ND	POS	L	ND	POS	L	ND	4 resp specimens: one pos by Xpert (rif not detected); two pos by culture (rif and inh susceptible on LPA)

(Footnotes for Table S2)

PID: participant identifier; S1: first stool specimen; Xp 06: Xpert 0.6g protocol; res: result; rif: rifampicin resistance result; semiq: semiquantitative Xpert result; Xp Sw: Xpert swab protocol; POS: positive; NEG: negative; resp: respiratory; VL: very low; ND: not detected; IND: indeterminate; inh: isoniazid; LPA: line-probe assay (MTBDRPlus); I/E: invalid or error result; L: low.

Table S5. Xpert positivity by different stool consistencies (per sample analysis)

**Stool consistency**	**Stool Xpert positive***		**Total**
	No (%)<br>n=398	Yes (%)<br>n=14	(%)<br>n=412
Liquid	38 (9.5)	0	38 (9.2)
Sticky	51 (12.8)	4 (28.6)	55 (13.3)
Semi-solid	206 (51.8)	8 (57.1)	214 (51.9)
Solid	103 (25.9)	2 (14.3)	105 (25.5)

**Includes any stool test on swab or 0.6g, from stool 1 and stool 2 if stool 2 was a different specimen from stool 1 (i.e. excludes second stool tests done on stool 1)*
